# P02.75. Outcome evaluation of the Veterans Affairs Salt Lake City Integrative Health Clinic for chronic nonmalignant pain and stress

**DOI:** 10.1186/1472-6882-12-S1-P131

**Published:** 2012-06-12

**Authors:** S Smeeding

**Affiliations:** 1 VA Salt Lake City Health Care System, Salt Lake City, USA

## Purpose

The purpose of this longitudinal outcome study was to determine the effectiveness of the Integrative Health Clinic and Program (IHCP) and to perform a subgroup analysis investigating patient benefit. The IHCP is the first organized integrative health clinical service within the Veterans Affairs Health Care System utilizing research based Complementary and Alternative Medicine (CAM), mind-body skills, and conventional treatments based on a health promotion and wellness model for the bio-psychosocial management of chronic non-malignant pain and stress related depression, anxiety, and symptoms of PTSD.

## Methods

A post hoc quasi experimental design was used combined with subgroup analysis to determine who benefited the most from the program. Data were collected at intake and up to four follow-up visits over a two year time period. Hierarchical Linear Modeling (HLM) was used for the statistical analysis and the outcome measures included: Health Related Quality of Life (SF-36), the Beck Depression Inventory (BDI) and Beck Anxiety Inventory (BAI). Comparisons included mental health subgroups and chronic nonmalignant pain subgroups.

## Results

The mental health subgroups with the greatest improvement, seen at 6 months, were found in the high anxiety group (Cohen’s d=.52), the high depression group (Cohen’s d=.46), and the PTSD group (Cohen’s d=.41). The chronic pain group with the greatest improvement at 6 months was the Chronic Non Spinal Pain group (joint pain, headache, fibromyalgia) with a decrease in depression and anxiety and improvement in health related quality of life at six months’ follow-up (Cohen’s d=0.74, 0.53, and 0.69, respectively); the benefit persisted over 24 months.

## Conclusion

This study provides evidence that the VA Integrative Health Clinic offers an effective reduction in pain-related psychopathology (e.g., depression and anxiety) and may improve some aspects of health related quality of life through use of nonpharmacological therapies, mind body skills and complementary and alternative Medicine.

